# NK cells promote neutrophil recruitment in the brain during sepsis-induced neuroinflammation

**DOI:** 10.1038/srep27711

**Published:** 2016-06-08

**Authors:** Hao He, Tingting Geng, Piyun Chen, Meixiang Wang, Jingxia Hu, Li Kang, Wengang Song, Hua Tang

**Affiliations:** 1 Institute of Immunology, Taishan Medical University, Taian, Shandong, 271000, China

## Abstract

Sepsis could affect the central nervous system and thus induces neuroinflammation, which subsequently leads to brain damage or dysfunction. However, the mechanisms of generation of neuroinflammation during sepsis remain poorly understood. By administration of lipopolysaccharides (LPS) in mice to mimic sepsis, we found that shortly after opening the blood–brain barrier, conventional CD11b^+^CD27^+^ NK subset migrated into the brain followed by subsequent neutrophil infiltration. Interestingly, depletion of NK cells prior to LPS treatment severely impaired neutrophil recruitment in the inflamed brain. By *in vivo* recruitment assay, we found that brain-infiltrated NK cells displayed chemotactic activity to neutrophils, which depended on the higher expression of chemokines such as CXCL2. Moreover, microglia were also responsible for neutrophil recruitment, and their chemotactic activity was significantly impaired by ablation of NK cells. Furthermore, depletion of NK cells could significantly ameliorate depression-like behavior in LPS-treated mice. These data indicated a NK cell-regulated neutrophil recruitment in the blamed brain, which also could be seen on another sepsis model, cecal ligation and puncture. So, our findings revealed an important scenario in the generation of sepsis-induced neuroinflammation.

During sepsis, the CNS is one of the first organs affected^1^. This is clinically manifested as sepsis-associated encephalopathy (SAE), characterized by cognitive impairment from mild delirium to deep coma, in 8–70% of septic patients^2^,^3^. Sepsis-induced neuroinflammation is thought to be the initial factor that contributes to CNS disorder and may affect neurotransmitters^4^,^5^. However, the mechanisms of generation of sepsis-induced neuroinflammation remain poorly understood.

Recent evidence showed that NK cells play an important role in sepsis^6^. In the model of cecal ligation and puncture (CLP), mice with NK cell depletion were protected against sepsis-induced mortality^7^. This is associated with the migration of NK cells from blood and spleen to the inflamed peritoneal cavity, where they promote the proinflammatory activities of myeloid cell populations^8^. For patients with septic shock, higher cytotoxity of NK cells led to higher mortality and worse organ function^9^.

How do NK cells contribute to sepsis-induced systemic inflammation? Crosstalk with other immune cells has been suggested^10^,^11^,^12^,^13^. Specifically, NK cells have been found to interact with neutrophils, the most abundant cell population in blood^14^. Recent findings showed that NK cells could promote neutrophils’ function and survival in co-culture system *in vitro*^15^,^16^. By using NK-cell-deficient mice, it has been suggested that NK cells promote neutrophil migration to the inflamed tissues such as liver, joints and lung^14^. However, the conclusion does not apply to the inflammation occurring in certain tissues such as colon^17^. For patients with sepsis, neutrophils can be detected in the cerebrospinal fluid^18^, but little is known about the migratory mechanism of neutrophils towards the CNS, and whether NK cells contribute to this process also remains unclear.

Although NK cells have been reported to be involved in some disorders of CNS such as infection, tumor, autoimmune encephalomyelitis, and exert neurotoxicity or neuroprotection^19^, their contribution to sepsis-induced neuroinflammation still enigmatic. In the animal model to mimic sepsis, we here observed the regulation of inflammation by NK cells in the brain, where CNS-infiltrated NK cells not only recruited neutrophils by producing chemokines but also promote neutrophil recruitment via modulating microglia. Furthermore, we found that depletion of NK cells could ameliorate depression-like behavior in mice with sepsis-induced neuroinflammation. This helps to understand the pathogenesis of SAE.

## Results

### NK cells infiltrated into the brain during LPS-induced neuroinflammation

**Figure 1:**
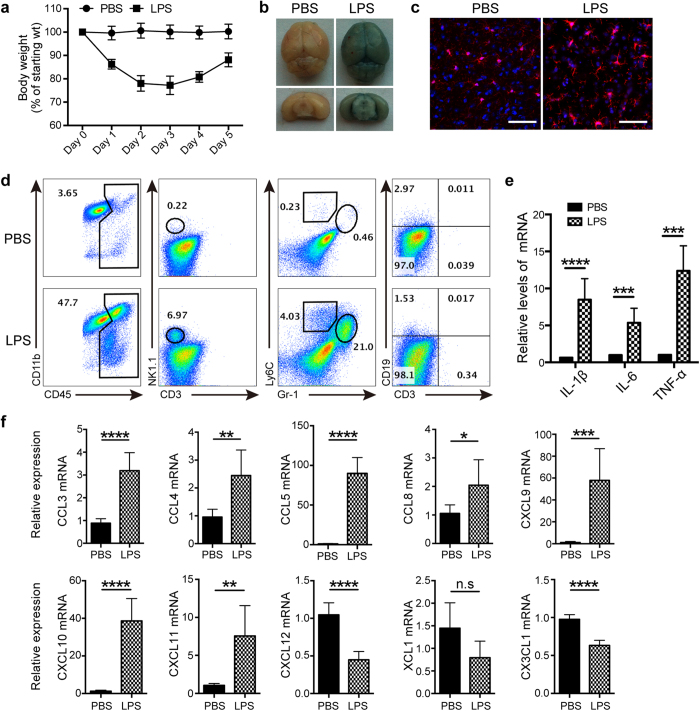
NK cells infiltrate into the brain after LPS treatment. (**a**) C57BL/6 mice were injected i.p. of LPS (2 mg/kg/day) diluted in PBS for 5 days, and the body weight was measured (n = 5~6 at indicated time). Mice treated only with PBS acted as the control (n = 6). (**b**) Mice-treated with PBS or LPS for 3 days received i.v. 0.1 ml of 4% Evans Blue perfusion. One hour later, mice were killed and the whole brain as well as coronal section were prepared to evaluate Evans Blue extravasation. (**c**) Following administration of PBS or LPS for 3 days, morphology of microglia in the brain was analyzed by immunofluorescence staining with Iba-1 (red) and DAPI (blue). Bars, 100 μm. (**d**) After 3 days of PBS or LPS treatment, single cell suspensions from the whole brain were prepared and analyzed by flow cytometry. CD45^+^ leukocytes were gated and analyzed for identification of cell types. (**e,f**) Twelve hours after LPS or PBS treatment, mRNA were extracted from brain of mice (n = 6 per group). qPCR was performed to detect the expression of proinflammatory cytokines and chemokines. *P < 0.05, **P < 0.01, ***P < 0.001, ****P < 0.0001, unpaired Student’s t test. Means ± SD are shown. (**b–d**) Data shown are representative of 4 mice per group. All data in this figure are representative of 3 independent experiments.

To mimic infection-induced sepsis, we administered LPS daily and found that the body weight of mice continuously declined, reaching the lowest point on day 2~3 followed by a recovery (Fig. 1a), which reflected the changes of systemic inflammation. To investigate whether LPS-induced inflammation spread into the CNS, we first examined the integrity of blood–brain barrier (BBB) by using Evans Blue i.v. injection. As shown in Fig. 1b, the brain was obviously stained blue after 3 days of LPS treatment, representing an extravasation of Evans Blue from blood, and indicating a disruption of BBB. Meanwhile, analysis by confocal microscopy showed that microglia from LPS-treated mice displayed active morphology, with larger cell bodies, retraction of branches, and stout or amoebic-like shapes when compared with those from PBS-treated controls (Fig. 1c). Activation of microglia is often considered as a marker for neuroinflammation, and simultaneous infiltration of CD45^hi^ leukocyte in the brain gave another evidence (Fig. 1d). These infiltrated leukocytes included CD19^−^CD3^−^NK1.1^+^ NK cells, CD11b^+^ Gr-1^hi^Ly6C^+^ neutrophils, CD11b^+^Gr-1^low^Ly6C^hi^ monocytes, but not CD3^+^ T lymphocytes and CD19^+^ B lymphocytes (Fig. 1d). It is not surprised that a large number of neutrophils migrated into the inflamed CNS, but obvious infiltration of NK cells into the brain greatly attracts our attention. In fact, 12 hours after LPS treatment when obvious leukocyte infiltration did not happen, expression of proinflammatory cytokines including IL-1β, IL-6 and TNF-α has been significantly upregulated in the brain (Fig. 1e). At this time, it was worth noting that 7 different chemokines for attracting NK cells were highly expressed in the brain (Fig. 1f). Similarly, we also observed the upregulation of certain chemokines for recruiting neutrophils (Supplementary Fig. 1). This gave a clue to explain why NK cells migrated into the CNS.

### NK cell-depletion alleviated LPS-induced neuroinflammation

**Figure 2:**
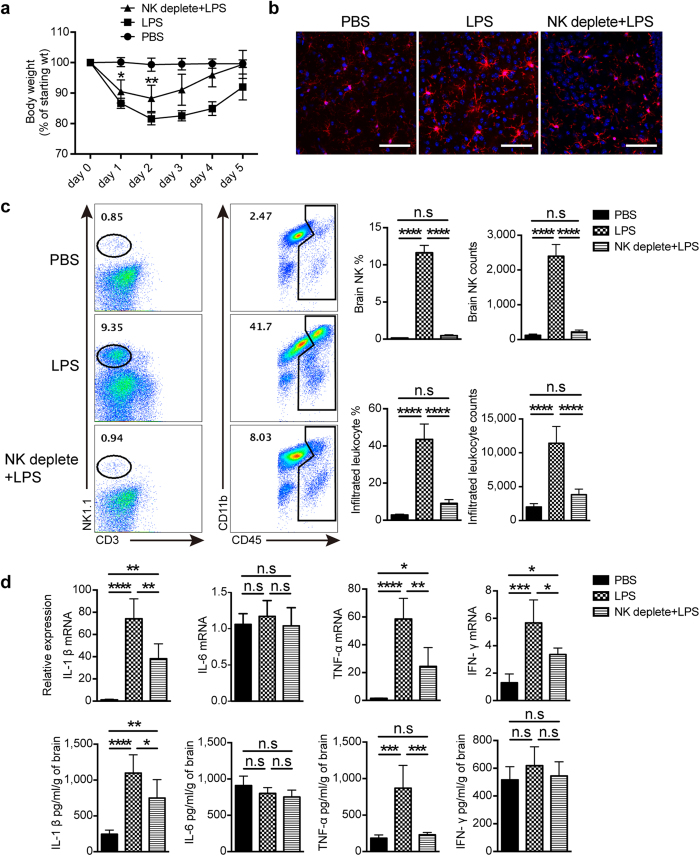
Depletion of NK cells alleviates LPS-induced neuroinflammation. Mice were injected i.p. with 25 μg of anti-NK1.1 Ab (PK136) once a day for 2 days to deplete NK cells, followed by a continuous LPS or PBS i.p. stimulation. (**a**) At indicated time point after PBS or LPS treatment, body weight of mice with or without NK cell depletion was measured (n = 6, per group). *P < 0.05, **P < 0.01, unpaired Student’s t test, when compared with LPS-treated group. Means ± SD are shown. (**b**) After 3 days of PBS or LPS treatment, immunofluorescence staining with Iba-1 (red) and DAPI (blue) was used to detect microglia morphology. Bars, 100 μm. Data shown are representative of 4 mice per group. (**c**) Following administration of PBS or LPS for 3 days, prepared single cell suspensions from the whole brain (n = 5 per group) were stained with indicated antibodies and analyzed by flow cytometry. CD45^+^ cells were gated to analyze CD19^−^CD3^−^NK1.1^+^ NK cells and CD45^hi^ infiltrated leukocytes. Histograms represent the percentage and cell number counted by flow cytometry. (**d**) Three days after PBS or LPS treatment, mRNA (n = 4 per group) and proteins (n = 5 per group) were extracted from brain of mice with or without NK cell-depletion. qPCR and ELISA were performed to detect the expression of proinflammatory cytokines. (**c,d**) *P < 0.05, **P < 0.01, ***P < 0.001, ****P < 0.0001, ANOVA. Means ± SD are shown. All data in this figure are representative of 3 independent experiments.

To understand the contribution of NK cells to LPS-induced neuroinflammation, anti-NK1.1 Ab was used to ablate NK cells prior to LPS treatment. We first found a significant accelerated recovery of body weight from day 1 in LPS-treated mice if NK cells had been depleted (Fig. 2a). This meant that depletion of NK cells protected mice from systemic inflammation, which also can be seen in CLP-induced sepsis as described below. We next explored the effect of NK cells on neuroinflammation, and observed the morphologic transformation of microglia from activation state on day 3 in LPS-treated mice into resting-like state in the lack of NK cells (Fig. 2b). Our data suggested a NK cell-regulated neuroinflammation, which was further supported by the evidence that not only percentage but also cell number of CD45^hi^ infiltrated leukocytes significantly decreased in the brain from mice treated with LPS for 3 days under the condition of NK cell depletion (Fig. 2c). We additionally detected mRNA and protein expression of proinflammatory cytokines in the brain from different group by qPCR and ELISA. As shown in Fig. 2d, LPS treatment strongly promoted the production of IL-1β and TNF-α, whose upregulation could be significantly inhibited by depleting NK cells. So, our data strongly suggested that NK cells play a critical role in determining the severity of neuroinflammation.

### NK cells controlled the infiltration of neutrophils into the brain during LPS-induced neuroinflammation

**Figure 3:**
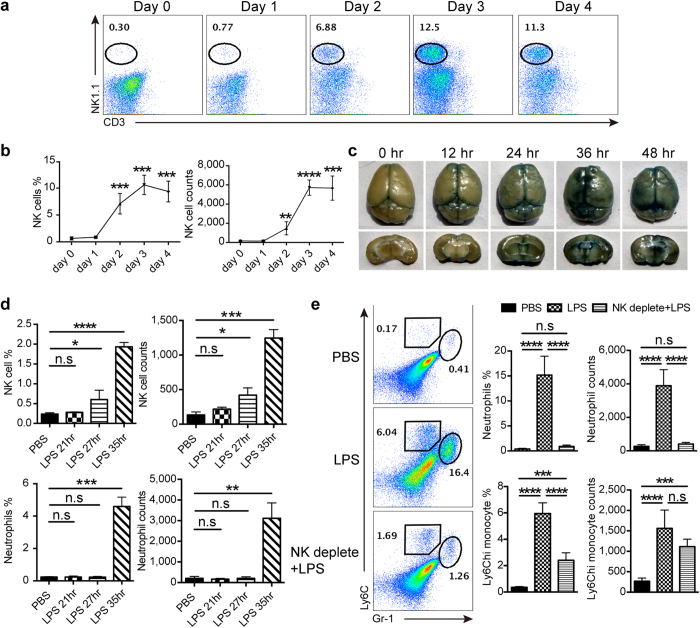
NK cells modulate neutrophil infiltration into the brain during LPS-induced neuroinflammation. (**a,b**) At indicated time point after LPS treatment, mice (n = 4 per group) were killed for assessment of NK cell infiltration in the brain. Histogram shows the percentage and cell number of CD19^−^CD3^−^NK1.1^+^ NK cells gated in CD45^+^ leukocytes. *P < 0.05, **P < 0.01, ***P < 0.001, ****P < 0.0001, unpaired Student’s t test, when compared with day 0. Means ± SD are shown. (**c**) Mice received 0.1 ml of 4% Evans Blue perfusion at different time points after LPS treatment. One hour later, the whole brain along with coronal sections were prepared to detect Evans Blue extravasation. Data shown are representative of 4 mice per group. (**d**) During LPS-induced neuroinflammation, single cell suspensions from the whole brain were prepared at indicated time points (n = 3 per group) and used for detection of NK cell (CD19^−^CD3^−^NK1.1^+^) and neutrophil (CD11b^+^Gr-1^hi^Ly6C^+^) infiltration. Histograms represent the percentage and cell number counted by flow cytometry. *P < 0.05, **P < 0.01, ***P < 0.001, ****P < 0.0001, unpaired Student’s t test, when compared with PBS-treated control. Means ± SD are shown. (**e**) Mice received anti-NK1.1 Ab (25 μg, twice) to deplete NK cells prior to administration of LPS. After 3 days of PBS or LPS treatment, mice (n = 5 per group) were killed and flow cytometry was used to analyze the percentage and cell number of Gr-1^hi^Ly6C^+^ neutrophils and Gr-1^low^Ly6C^hi^ monocytes gated in CD45^+^ leukocytes. ***P < 0.001, ****P < 0.0001, ANOVA. Means ± SD are shown. All data in this figure are representative of at least 2 independent experiments.

As NK cells have been observed to act as an important player in LPS-induced neuroinflammation, we are interested in how these cells work during the process. We first dynamically monitored the NK cell infiltration in the brain. As shown in Fig. 3a,b, an obvious increase in the percentage and cell number of NK cells appeared in the brain from day 2 after LPS treatment, suggesting an opening of BBB within 48 hours. We thus performed Evans Blue extravasation assay to examine when BBB integrity was damaged, and found that the dye started to diffuse into the brain between 12 and 24 hours after receiving LPS (Fig. 3c). Subsequent experiments were designed to explore the sequence of immune cell infiltration in the brain in the early stage of BBB disruption. As displayed in Fig. 3d, it took about 27 hours for NK cells to obviously infiltrate into the brain after LPS administration, whereas neutrophil migration could not be observed until 35 hours passed. This indicated that NK cells arrived at the brain prior to neutrophils. Considering the crosstalk between NK cells and neutrophils found in other disease models^14^, we supposed that NK cells might modulate neutrophil migration during neuroinflammation. Experiments were so performed in LPS-treated mice, and the result showed that there was a significant decline in the percentage and cell number of neutrophils and monocytes in the brain once NK cells had been depleted (Fig. 3e). This supported our hypothesis about NK cell-regulated neutrophil recruitment in the generation of neuroinflammation.

### Brain-infiltrated NK cells attracted neutrophils in chemokine-dependent manner

**Figure 4:**
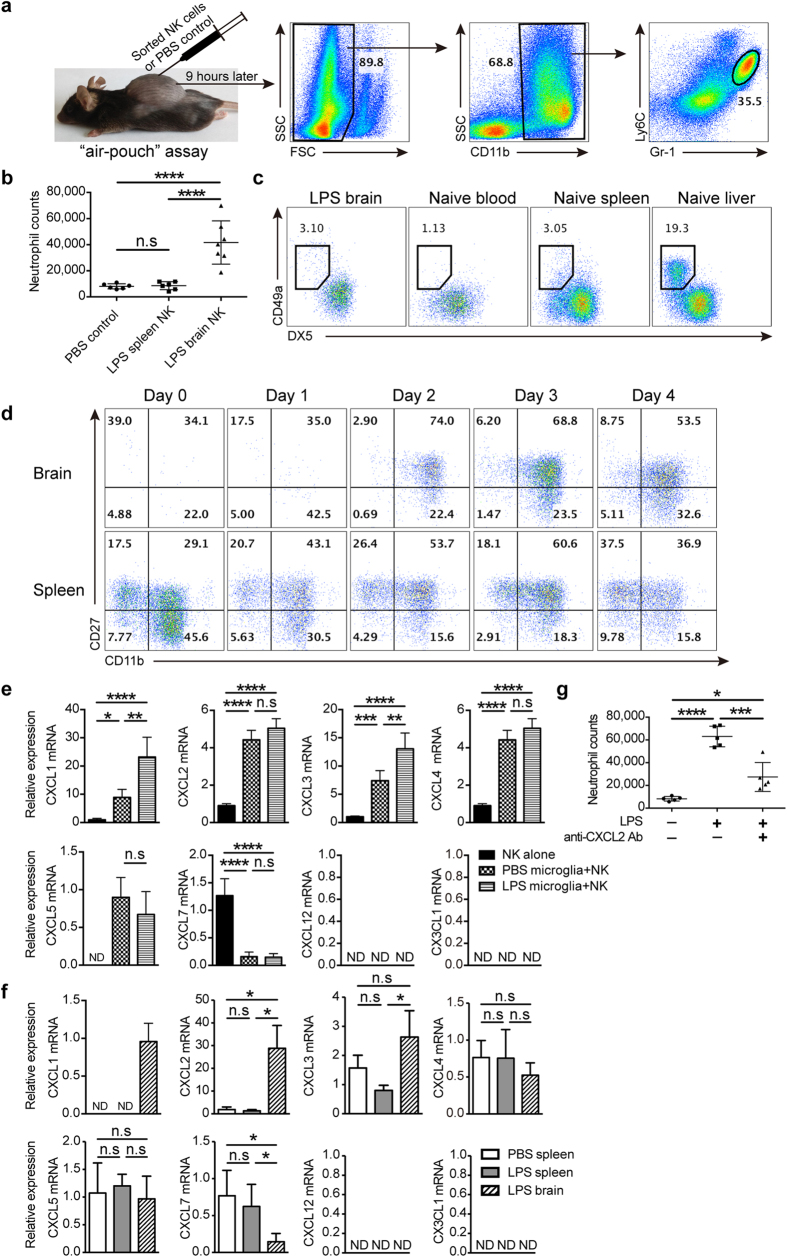
Brain-infiltrated NK cells attract neutrophils by producing chemokines during LPS-induced neuroinflammation. (**a**) Performance of *in vivo* recruitment assay, i.e., air pouch assay. NK cells (8 × 10^4^) sorted by flow cytometry from brain or spleen were injected into the air pouch on the back of naïve mice. Nine hours later, cells were obtained from the air pouch and CD11b^+^Gr-1^hi^Ly6C^+^ neutrophils were counted by flow cytometry. (**b**) Scatter plot showed the cell number of neutrophils attracted into the air pouch (n = 6~7, per group) by sorted NK cells from the brain and spleen in mice experiencing LPS stimulation for 3 days. (**c,d**) Single cell suspensions were prepared from the brain, spleen, blood, and liver in PBS-treated mice or LPS-treated mice, followed by CD19^−^CD3^−^NK1.1^+^ NK cell phenotype analysis via flow cytometry. Data shown are representative of 4 mice per group. (**e**) CD19^−^CD3^−^NK1.1^+^ NK cells (1 × 10^5^) sorted from bone marrow in naïve mice were cocultured with or without microglia (2 × 10^5^) sorted from mice treated with PBS or LPS for 3 days. Eleven hours later, NK cells in the coculture were sorted by flow cytometry again for mRNA extraction and subsequent chemokine analysis by qPCR. *P < 0.05, **P < 0.01, ***P < 0.001, ****P < 0.0001, ANOVA. Means ± SD are shown. (**f**) mRNA was extracted from NK cells (1.5 × 10^4^) that were sorted from brain and spleen in mice treated with PBS or LPS for 3 days (n = 4~5, per group). qPCR was performed to assess chemokine expression. (**g**) Air pouch assay was performed in mice (n = 5, per group) to detect the chemotaxis of brain-infiltrated NK cells (8 × 10^4^) that derived from mice treated with LPS for 3 days in the presence of CXCL2 blocking Ab (1μg/ml). (**b,e–g**) *P < 0.05, **P < 0.01, ***P < 0.001, ****P < 0.0001, ANOVA. Means ± SD are shown. All data in this figure are representative of at least 2 independent experiments.

We wondered how NK cells modulate neutrophil infiltration, and air pouch assay was performed to test the chemotactic activity of NK cells *in vivo* (Fig. 4a). The result showed that brain-derived, but not spleen-derived, NK cells from LPS-treated mice exhibited activity to recruit neutrophils (Fig. 4b). This indicated that NK cells located in the brain and spleen, even from the same LPS-treated mouse, have different function. To investigate whether different NK cell subsets led to this discrepancy in chemotaxis, we compared the phenotype of NK cells in the brain and spleen. The result showed that NK cells in the brain belonged to conventional DX5^+^CD49a^−^ NK cell subset similar to that in the blood and spleen, but distinguished from the subset in the liver, where a unique resident DX5^−^CD49a^+^ NK cell subset was observed^20^,^21^ (Fig. 4c). Another method to classify NK cell subsets based on maturation stage by the expression of CD11b and CD27^22^, was also used. Through dynamic monitoring of NK cell infiltration, we found that CD11b^+^CD27^+^ NK cell subset initially infiltrated into the brain after LPS treatment and constituted the main body of NK cells thereafter. Similarly, this subset also represented the largest proportion of NK cells in the spleen (Fig. 4d). So, difference in NK cell subsets seemed not to interpret the different chemotactic activity of NK cells between brain and spleen. We next investigated whether this was attribute to the education by tissue microenvironment. As shown in Fig. 4e, after coculture *in vitro* for 11 hours with microglia from naïve mice, bone marrow-derived naïve NK cells upregulated mRNA of neutrophil-attracting chemokines, such as CXCL1, CXCL2, CXCL3, CXCL4 and CXCL5. If microglia were from mice experienced LPS stimulation for 21 hours when NK cells would soon migrate into the brain, cocultured NK cells expressed much higher level of CXCL1 and CXCL3 mRNA. We also observed that microglia could educate NK cells to upregulate proinflammatory cytokines, including IL-1β, IL-6, TNF-α and IFN-γ (Supplementary Fig. 2). These data indicated that microglia, an important component of CNS microenvironment, could act as an educator to affect the function of NK cells.

To further confirm the education provided by tissue microenvironment *in vivo*, we extracted mRNA from NK cells sorted from the brain and spleen by flow cytometer to compare chemokine expression. As shown in Fig. 4f, brain-infiltrated NK cells expressed more CXCL1, CXCL2 and CXCL3, but not CXCL4, CXCL5 and CXCL7, when compared with NK cells in the spleen from LPS or PBS-treated mice. Different chemokine profiles of NK cells between the brain and spleen thus supported the education of NK cells by tissue microenvironment. As chemokines are closely associated with chemotaxis, we thus evaluated the importance of chemokines for NK cells to attract neutrophils, and selected CXCL2 as the target considering its significant upregulation in the brain. As predicted, attraction of neutrophils by CNS-infiltrated NK cells was significantly impaired after neutralizing CXCL2 in the air pouch (Fig. 4g). Taking CXCL2 as the example, we confirmed that attraction of neutrophils by brain-infiltrated NK cells depended on chemokine production, which were greatly affected by tissue microenvironment.

### NK cells regulated microglia to recruit neutrophils during LPS-induced inflammation

**Figure 5:**
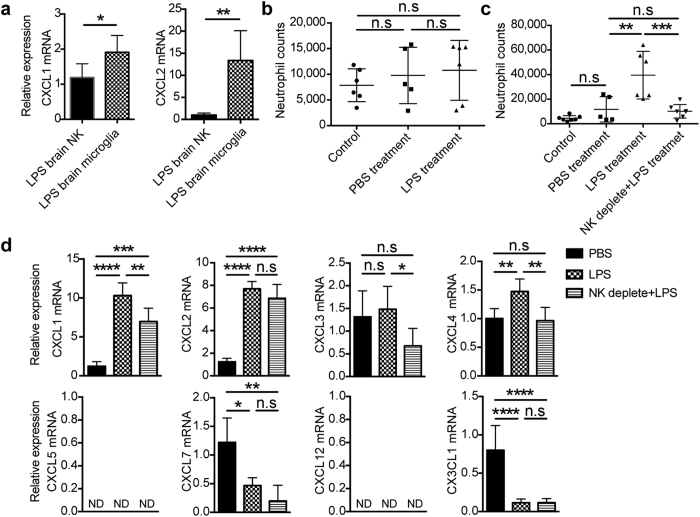
NK cells regulate microglia to recruit neutrophils during LPS-induced inflammation. (**a**) Three days after LPS treatment, CD19^−^CD3^−^NK1.1^+^ NK cells (1.5 × 10^4^) and CD3^−^CD19^−^NK1.1^−^Gr-1^−^Ly6C^−^CD11b^+^CD45^+^ microglia (1.5 × 10^4^) were sorted from brain (n = 5) by flow cytometry for analysis of chemokine mRNA expression via qPCR. *P < 0.05, **P < 0.01, unpaired Student’s *t* test. Means ± SD are shown. (**b**) Sorted microglia (8 × 10^4^) from PBS-treated mice (21 hours, n = 5) and LPS-treated mice (21 hours, n = 6) were used to detect the attraction of neutrophils by air pouch assay. (**c**) Microglia (8 × 10^4^) were purified from PBS-treated mice (3 days, n = 5) and LPS-treated mice (3 days, n = 6) with or without NK cell depletion. Air pouch assay was performed to detect the chemotaxis of neutrophils by microglia from different groups. (**d**) Following administration of PBS or LPS for 3 days, microglia (1.5 × 10^4^) were sorted from mice with or without NK cell-depletion for mRNA extraction and subsequent chemokine analysis by qPCR (n = 4~6, per group). (**b–d**) *P < 0.05, **P < 0.01, ***P < 0.001, ****P < 0.0001, ANOVA. Means ± SD are shown. All data in this figure are representative of 3 independent experiments.

Besides directly attracting neutrophils, NK cells might indirectly modulate neutrophil recruitment by crosstalk with other immune cells especially the CNS-resident microglia, as tissue resident cells have the potential to guide neutrophils to the inflamed site^23^. From analysis of chemokine expression by qPCR, we observed that sorted microglia from LPS-treated mice had an even significant higher expression of CXCL1 and CXCL2 compared with brain-infiltrated NK cells (Fig. 5a), suggesting a potential of microglia to recruit neutrophils. We thus evaluated the chemotactic activity of microglia in LPS-treated mice using *in vivo* recruitment assay. As shown in Fig. 5b, 21 hours after LPS treatment when NK cells would soon migrate into the CNS and did not encounter with microglia yet, sorted microglia did not display chemotaxis to neutrophils compared with that from PBS-treated mice (Fig. 5b). However, we found a much stronger recruitment of neutrophils by microglia from brain with obvious NK cell infiltration in mice treated with LPS for 3 days (Fig. 5c). Before and after the infiltration of NK cells, microglia clearly displayed different chemotactic activity. This promoted us to study the relation between microglia chemotaxis and NK cell infiltration. We thus compared the expression of chemokines for attracting neutrophils on microglia sorted from PBS and LPS-treated mice. As shown in Fig. 5d, microglia significantly upregulated CXCL1, CXCL2, CXCL4 but not CXCL3, CXCL7 and CX3CL1 mRNA expression after 3 days of LPS treatment. However, NK cell depletion significantly reduced the expression of CXCL1, CXCL3 and CXCL4 on microglia (Fig. 5d). Different chemokine profiles displayed by microglia in the presence or absence of NK cell infiltration reflected the functional changes, which also supported by the alteration of activation state (Fig. 2b). Importantly, once NK cells had been depleted, microglia from LPS-treated mice displayed impaired chemotaxis to neutrophils (Fig. 5c). So, our data indicated a microglia-mediated pathway for attraction of neutrophils, which was also regulated by NK cells.

### NK cells regulated neutrophil recruitment into the brain during CLP-mediated neuroinflammation

**Figure 6:**
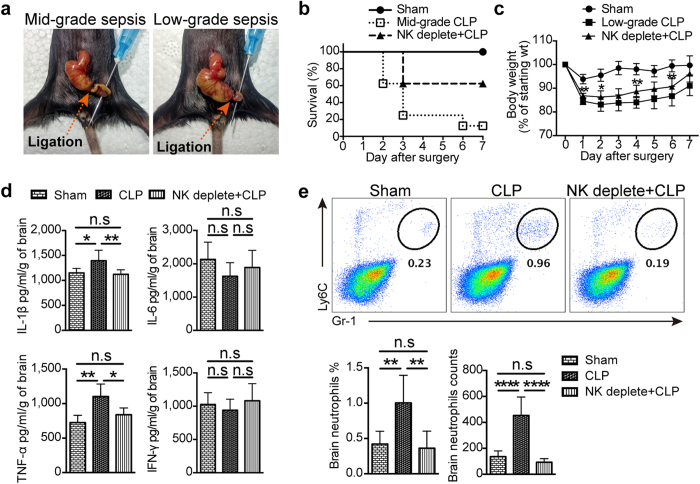
NK cells regulate neutrophil recruitment in the brain during CLP-induced neuroinflammation. (**a**) Two CLP models with different severity were prepared. For mid-grade CLP, 50% of the cecum was ligated; For low-grade CLP, 25% of the cecum was ligated. (**b**) Survival rate in sham group, mid-grade CLP group with or without NK cell depletion (n = 8, per group) was detected in 7 days after surgery. (**c**) The body weight of mice (n = 8 per group) in 7 days after surgery was measured in mice with low-grade CLP. *P < 0.05, **P < 0.01, unpaired Student’s t test, when compared with low-grade CLP group. Means ± SD are shown. (**d**) Three days after surgery, proteins were extracted from the whole brain of mice in sham group, low-grade CLP group with or without NK cell depletion (n = 6 per group). ELISA was performed to detect the concentration of proinflammatory cytokines in the brain. (**e**) Three days after surgery, prepared single cell suspensions from the whole brain in low-grade CLP group and control group (n = 5 per group) were stained and analyzed by flow cytometry. Histogram shows the percentage and cell number of CD45^+^Gr-1^hi^Ly6C^+^ neutrophils. (**c–e**) *P < 0.05, **P < 0.01, ***P < 0.001, ****P < 0.0001, ANOVA. Means ± SD are shown. All data in this figure are representative of at least 2 independent experiments.

Although NK cell-promoted neutrophil recruitment in the brain has been observed in LPS-induced neuroinflammation, we wonder whether we could confirm this phenomenon on another sepsis model—CLP. When compared with LPS treatment, the CLP model is more realistic for the induction of polymicrobial sepsis in experimental settings, thus suitable for verifying the contribution of NK cells to sepsis-induced neuroinflammation. We thus prepared two CLP models with different severity, which depended on the length of ligated cecum (Fig. 6a). In mid-grade CLP, NK cell depletion obviously increased the survival rate from less than 20% to about 65% (Fig. 6b), indicating the key role of NK cells in the regulation of sepsis-induced systemic inflammation. However, mid-grade CLP is not suitable to investigate the generation of neuroinflammation, considering the inconsistence in time of death. So, low-grade CLP was used, as mice with this model displayed mild sepsis characterized by reversible body-weight decline but no death (Fig. 6c). Similar to mid-grade CLP model, depletion of NK cells significantly promoted the recovery of body weight in low-grade CLP (Fig. 6c), confirming the regulatory activity of NK cells during sepsis. Furthermore, we found a significant increase in the concentration of IL-1β and TNF-α in the brain of mice with low-grade CLP 3 days after surgery (Fig. 6d), indicating an infection-initiated neuroinflammation. More importantly, depletion of NK cells prior to surgery could significantly inhibit these two cytokines production in the brain (Fig. 6d). This supported the modification of neuroinflammation by NK cells just like that in LPS-treated mice. Consistent with the upregulation of proinflammatory cytokines in the brain, obvious neutrophil infiltration was also observed in the CNS 3 days after surgery, which could be significantly restrained by depleting NK cells (Fig. 6e). Thus, by using CLP-induced sepsis model, we again confirmed the regulation of neutrophils by NK cells in the CNS, which constituted an important scenario during the generation of sepsis-induced neuroinflammation.

### NK cells affected depression-like behavior during sepsis-induced neuroinflammation

**Figure 7:**
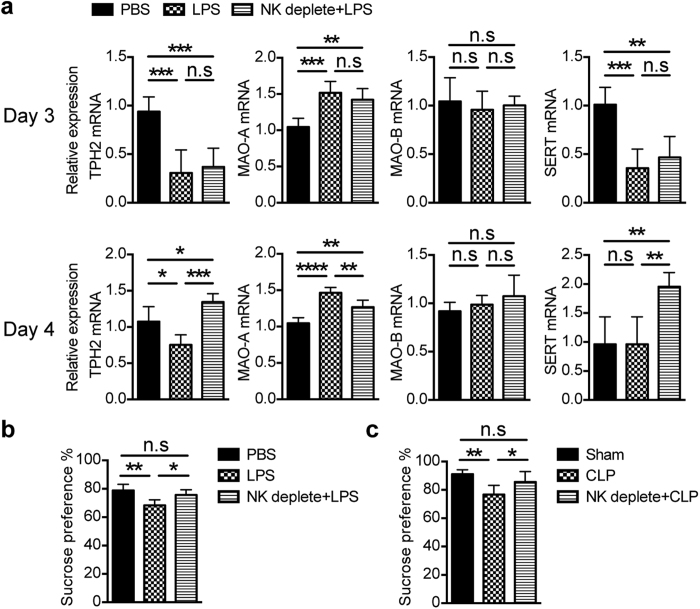
NK cells regulate depression-like behavior during sepsis-induced neuroinflammation. Mice with or without depletion of NK cells were treated with LPS or received surgery. (**a**) mRNA was extracted from the whole brain (n = 4~6 per group) on day 3 and day 4 after LPS treatment and reversely transcribed into cDNA, then qPCR was performed to detect and analyze the expression of serotonin metabolism-associated enzymes and proteins, including tryptophan hydroxylase 2 (TPH2), aonoamine oxidase (MAO-A and MAO-B) and serotonin transporter (SERT). (**b,c**) Four days after LPS treatment or low-grade CLP surgery, sucrose preference test was performed to evaluate the depression-like behavior of mice from different group (n = 8, per group). (**a–c**) *P < 0.05, **P < 0.01, ***P < 0.001, ****P < 0.0001, ANOVA. Means ± SD are shown. All data in this figure are representative of at least 2 independent experiments.

Neuroinflammation is often companied with brain dysfunction, which frequently represents “sickness behaviors” including lethargy, anorexia, increased anxiety, depressed mood, apathy and so on. Some of these behaviors have been regarded as a response of neurons to cytokines in several animal models^24^,^25^,^26^,^27^,^28^. Given the fact that NK cell-depletion will affect cytokine production in the brain (Figs 2d and 6d), we postulate that CNS-infiltrated NK cells might have an effect on nervous system. By scanning the expression of rate-limiting enzymes for neurotransmitter metabolism using qPCR, we found significant changes on the expression of serotonin metabolism-associated enzymes and proteins including tryptophan hydroxylase 2 (TPH2), monoamineoxidase (MAO-A) and serotonin transporter (SERT) in the brain from mice treated with LPS for 3 days (Fig. 7a). Of interest, alteration of these molecules can be partially or completely restored on the following day through depletion of NK cells, suggesting a regulation of serotonin metabolism by NK cells (Fig. 7a). We also detected synthesis/degradation-associated rate-limiting enzymes for other neurotransmitters, but the data seemed not to support their regulation by NK cells (Supplementary Fig. 3). As serotonergic system is well known to contribute to depression^29^, this drives us to evaluate the effect of NK cells on depression-like behavior in mice. Sucrose preference test was therefore performed to evaluate anhedonia, a core symptom of major depression^30^. We found a reduced sucrose preference in LPS-treated mice when compared with controls (Fig. 7b), reflecting a depression-like behavior reported by previous studies^31^,^32^. Interestingly, NK cell depletion significantly ameliorated this depression-like behavior in LPS-treated mice (Fig. 7b), which also can be seen in low-grade CLP model (Fig. 7c). However, regulation of depression-like behavior by NK cells only can be seen at certain period of disease. As severity of depression-like behavior changed with time, we did not observed this regulation on day 2 and day 7 after surgery in low-grade CLP (Supplementary Fig. 4). Anyhow, we get an insight into the “bridge” to connect neuroinflammation to sickness behaviors on the cellular level.

## Discussion

SAE, as the most common cause of encephalopathy in the intensive care unit, is characterized by diffuse brain dysfunction that occurs secondary to sepsis in the body without overt CNS infection^2^. Sepsis-induced neuroinflammation is the core of SAE, but how it gradually occurs still remains poorly understood. By administration of LPS or preparation of CLP to mimic sepsis^31^,^33^,^34^, we explored the generation of sepsis-induced neuroinflammation. After LPS treatment, we first detected the dynamic changes of NK cells and neutrophils in the blood and brain, and found that not only percentage but also cell count of NK cells rapidly decreased in the blood followed by a slow rebound. Meanwhile, NK cells increased in the brain (Supplementary Fig. 5). This may reflect the migration of NK cells from blood to inflamed tissues such as brain, as well as a supplement of NK cells in the blood from the bone marrow. For neutrophils, the tendency of dynamic changes was similar to NK cells in the brain but not in the blood (Supplementary Fig. 5). This difference may reflect more rapid cell renewal of neutrophils from the bone marrow. However, there was still controversy about the role of NK cells in sepsis in patients^6^,^35^. Discrepancies concerning the number and/or function of circulating NK cells are probably due to the heterogeneity of patients in term of either severity (severe sepsis and/or septic shock) or involvement of pathogens (Gram-negative versus positive bacteria)^36^. In animal model, it is interesting that NK cells were observed accumulated in the inflamed brain prior to neutrophil migration. We do not clearly know the cause of NK cell infiltration, but a previous report gives a clue that PMA-stimulated microglia could recruit CNS-derived NK cells sorted from mice with autoimmune encephalomyelitis *in vitro* via releasing monocyte chemotactic protein-1^37^. We thus sorted microglia from mice experienced LPS stimulation for 21 hours when NK cells would soon migrate into the brain, and evaluated their chemotaxis to NK cells by *in vivo* recruitment assay. Our data showed that microglia could not attract NK cells at that time (Supplementary Fig. 6), suggesting that microglia were not important for NK cell migration towards the CNS in the early stage of neuroinflammation. Other cell types such as cerebrovascular endothelial cells, astrocytes and neuron might be responsible for NK cell infiltration into the brain.

Our works mainly focus on the contribution of NK cells to the generation of sepsis-induced neuroinflammation. To our surprise, depletion of NK cells significantly ameliorates the LPS or CLP-induced neuroinflammation. This indicates a regulation of sepsis-induced neuroinflammation by NK cells, which has not been reported in previous studies. Through dynamic monitoring of NK cell infiltration, we found that CD11b^+^CD27^+^ NK cell subset initially infiltrates into the inflamed brain and constitutes the main body of NK cells. As expression level of CD11b and CD27 often reflect maturation stage^22^, our data suggests it is the mature NK cells that mainly participate in the regulation of neuroinflammation. Moreover, no DX5^−^CD49a^+^ NK cell subset was observed in the inflamed brain. This means that brain-derived NK cells are migratory, because DX5^+^CD49a^−^ NK cell subset but not DX5^−^CD49a^+^ NK cell subset have been demonstrated to have the ability to migrate *in vivo* by parabiotics detection technique^20^.

It is especially important that the migration of neutrophils towards the CNS is severely impaired once NK cells have been depleted. Thus, regulation of neutrophil recruitment in the brain by NK cells seemed to be an important scenario during the generation of neuroinflammation. This sparks our interest in how NK cells regulate neutrophil infiltration into the CNS. Taking advantage of *in vivo* recruitment assay^38^, we observed that brain-infiltrated NK cells had the ability to attract neutrophils in chemokine-dependent manner. It should be noted that chemotactic activity of brain-derived NK cells is affected by the education of tissue microenvironment, as microglia regardless of activation seemed to be sufficient to promote cytokine production by infiltrating NK cells. Our data only showed the changes of chemokine expression by NK cells in mRNA level but not protein level, considering the very small amount of NK cells in the inflamed brain. Additionally, we found that there just had a trend of increased IFN-γ at protein level in the inflamed brain. NK cell-dependent upregulation of IFN-γ may be masked by environmental noise, considering the rare NK cells in the CNS and the variety of source of IFN-γ from other immune cells. Altogether, we found a functional changes of NK cells affected by CNS microenvironment especially microglia, which had been suggested by previous study^37^. This still needs to be further confirmed *in vivo*.

Additionally, we found an alternative pathway to regulate neutrophil recruitment in the inflamed brain, that is, microglia-mediated attraction. Based on their large amount and wide distribution, microglia are thought to be an important CNS-resident immune cells^39^. Not only is activation of microglia a frequent marker to indicate neuroinflammation, but microglia also function as guards to eliminate microorganisms and protect neurons^40^,^41^. Our results confirmed the important role of microglia in LPS-induced neuroinflammation, as these cells became activated and exhibited chemotaxis to neutrophils. Additionally, chemotactic activity of microglia could be regulated by NK cells. So, during sepsis-induced neuroinflammation, brain-infiltrated NK cells not only directly attract neutrophils, but also indirectly promote neutrophil recruitment via modulating microglia. This finding is helpful to deeply understand the formation of neuroinflammtion.

So, what this means to the CNS? It is easy to connect neuroinflammation with brain dysfunction. Our data about LPS or CLP-induced depression-like behavior provided the evidence, and was consistent with previous reports^31^,^32^. However, what is the bridge to connect neuroinflammation to sickness behaviors? Considering the fundamental role of neurotransmitters in nerve signal transduction, we speculate that these signal molecules might act as the mediators to link neuroinflammation and behavioral change. Briefly, we proposed that infiltrated leukocytes promote inflammation in the brain, where increased proinflammatory cytokines affect serotonin metabolism and subsequent depression-like behavior. This hypothesis is partially supported by our data and other evidences from previous reports. We have demonstrated that NK cell depletion could significantly reduce IFN-γ and TNF-α production in the brain. These two inflammatory cytokines have been proven to be able to activate indoleamine 2,3 dioxygenase^42^,^43^,^44^, which plays an important role in LPS-induced depression-like behavior through degradation of tryptophan, the rate-limiting acid for serotonin synthesis^32^. Our findings that depletion of NK cells could ameliorate depression-like behavior in mice experienced neuroinflammation provides favorable evidence. So, cytokine–neurotransmitter–behavior axis seems to implicate in sepsis-induced brain dysfunction.

Taken together, our data indicated a NK cell-regulated neutrophil recruitment in the CNS, which constituted an important scenario during the generation of sepsis-induced neuroinflammation. This expands our understanding about the pathogenesis of SAE, but how CNS-infiltrated NK cells modulate microglia and regulate the metabolism of serotonin still needs further investigation.

## Methods

### Mice

Female C57BL/6 (B6; H-2 Kb) mice, 6–8 weeks of age, were purchased from Vitalriver (Beijing, China) and maintained in SPF condition. Study was approved by the Laboratory Animal Care Committee of Taishan Medical University, and all animal experiments were conducted in accordance with the Guidelines of Care and Use of Laboratory Animals at the Taishan Medical University.

### LPS administration and CLP model preparation

For LPS administration, Mice were injected i.p. daily of 2 mg/kg LPS (*E. coli*, serotype 0111:B4; Sigma-Aldrich) diluted in saline (PBS) for different days. Mice treated only with PBS were used as control. For CLP model preparation, the length of ligated cecum is primarily linked to the mortality and serum levels of proinflammatory cytokines^45^. Two different severity of CLP was performed as previously described^46^. For mid-grade CLP, 50% of the cecum was ligated, and mice were prone to death after surgery. For low-grade CLP, 25% of the cecum was ligated, and no death occurred in mice. In some experiments, mice were injected i.p. twice with 25 μg of anti-NK1.1 mAb (prepared from PK136 hybridoma culture supernatant) to deplete NK cells prior to LPS administration or CLP preparation. We also confirmed NK cell depletion by staining NKp46. More than 90% of NK cells were depleted.

### Preparation of single cell suspensions

For brain single cell preparation, mice received cardiac perfusion with 0.01 M PBS. The brains were removed and digested with 2 μg/ml collagenase II (Sigma-Aldrich) at 37 °C for 15 minutes followed by mixture with EDTA (0.02 M, Invitrogen) for 5 minutes. Tissues were filtered through a 70 μm cell strainer and were resuspended in 40% percoll (GE Healthcare, Uppsala, Sweden) for centrifugation at 1400 G for 20 minutes. Cell pellets at the bottom were collected for further analysis. Liver single cell suspension was prepared in mice receiving portal vein perfusion. The liver was filtered through a 200-gauge stainless steel mesh and subsequently resuspended in 40% Percoll. After centrifugation for 20 minutes at 1200 G, cell pellets at the bottom were collected and lysed with ACK Lysing buffer (Gibco Life Technologies). Splenocytes were isolated by forcing the spleen through stainless steel mesh and subsequently lysing erythrocytes. Blood leukocytes were purified by lysing erythrocytes with ACK Lysing buffer.

### Flow cytometry

For cell surface staining, prepared single cell suspensions were first blocked with anti-Fcγ III/II Receptor mAb (2.4 G2) for 10 minutes followed by staining with fluorescence-conjugated mAb for CD45 (30-F11), CD3 (145-2C11), NK1.1 (PK136), CD19 (1D3), CD11b (M1/70), Gr-1 (RB6-8C5), Ly6C (HK1.4), CD27 (LG.7F9), CD49b (DX5), CD49a (HMα1ch). Most of mAbs were purchased from eBioscience (San Diego, CA) except CD49a mAb (Biolegend, San Diego, CA). For NK cell sorting, CD3^−^CD19^−^NK1.1^+^ cells from CD45^+^ cells were gated as NK cells. To purify microglia, NK1.1^−^Gr-1^−^Ly6C^−^CD3^−^CD19^−^CD11b^+^ cells were sorted from CD45^+^ cells in the brain, as no very specific marker could be used to identify microglia. Briefly, CD45^+^ cells were first gated from tissue single cell suspension, then CD19^−^ cells were gated for sorting CD3^−^NK1.1^+^ NK cells; CD11b^+^ cells were next gated from the remaining CD3^−^NK1.1^−^ cells, and Ly6C^−^Gr-1^−^ cells were subsequently gated for sorting microglia. Purity of sorted cell population was more than 90%. For cell count, stained cells were collected at high speed for 40 seconds and counted by flow cytometer. Phenotypic analysis, cell sorting and cell count were performed on BD FACS Aria II Flow Cytometer.

### Tissue ELISA assay

After cardiac perfusion, the brain was removed and weighed. Following homogenization with homogenizor (OPTIMA INC, Japan) and lysation by 1 ml of RIPA Buffer (Cell Signaling Technology, Inc) per brain, proteins from brain were extracted after centrifugation. Concentration of IL-1β, IL-6, TNF-α and IFN-γ in the brain were determined by ELISA Ready-SET-Go Kit (eBioscience) according to the manufacturers’ protocol. Results were shown as concentration of cytokines per gram of brain.

### qPCR

Total RNA was extracted either from the whole brain using TRIzol (Invitrogen) or from sorted NK cells and microglia using RNeasy Mini Kit (Qiagen). First-strand cDNA of each sample was synthesized using QuantiTect Reverse Transcription Kit (Qiagen) according to the manufacturers’ instructions. cDNA was used as a template for quantitative realtime-PCR using SYBR Green Master Mix (Qiagen). PCR and analysis was performed on the Rotor-Gene Q (Qiagen). Gene expression was calculated relative to that of *gapdh*. Primers were showed in Supplementary Table S1.

### Immunohistofluorescent staining

After cardiac perfusion with 0.01 M PBS and subsequent 0.1 M PBS containing 4% Paraformaldehyde (PFA, Sigma-Aldrich), collected brain were post-fixed in 0.1 M PBS containing 4% PFA overnight at room temperature (RT) and embedded in OCT (SAKURA). For Iba-1 assay, sections measuring 20 μm were blocked with blocking buffer (0.4% Triton X-100, 4% normal goat serum and 1% BSA in 0.01 M PBS) for 1 hour at RT, followed by incubation with rabbit anti-mouse Iba-1 (Wako Chemicals) at 4 °C overnight. On the following day, sections were incubated with goat anti-rabbit Ig-PE (Santa Cruz) for 2 hours. After wash, sections were further incubated with DAPI (Invitrogen) for 5 minutes. Images were captured using spinning-disk confocal microscopy (Leica SD AF).

### Evans Blue extravasation

Mice were injected i.v. of 0.1 ml of 4% Evans Blue solution. One hour later, mice were anesthetized followed by cardiac perfusion with 0.01 M PBS and subsequent 4% PFA (Sigma-Aldrich). Fixed brains were removed. Photographs of the entire brain as well as coronal section were taken to assess Evans Blue extravasation.

### *In vivo* recruitment assay (“Air pouch” assay)

The subcutaneous “air-pouch” assay was used as previously described^38^. Briefly, air pouch were generated by injection of 3 ml air into the subcutaneous tissue on the back of anesthetized mice. Air pouch were reinflated 3 days later. On the next day, sorted 8 × 10^4^ NK cells or microglia were suspended in 0.5 ml of PBS and injected into the pouch. Mice received only 0.5 ml of PBS were used as controls. Nine hours later, cells obtained from air pouch were stained by anti-CD11b, Gr-1 and Ly6C mAb for cell count by flow cytometry. In certain recruitment assay, NK cells were mixed with 1 μg/ml CXCL2 blocking antibody (PeproTech) in the air pouch.

### Sucrose preference test

Mice experienced deprivation for 12 hours were given a choice between two bottles, one with 5% sucrose solution (wt/vol) and another with normal drinking water for 3 hours. Mice were tested over another 24 hours, during which bottle positions were switched every 12 hours to prevent the possible effects of side preference in drinking. The consumption of water and sucrose were measured by weighing the bottles. Sucrose preference was evaluated via the sucrose uptake rate, namely, the percentage of consumed sucrose-containing solution relative to the total amount of liquid intake.

### Statistical analysis

Statistical significance of differences was determined by Student’s *t* tests for 2 groups or ANOVA for 3–4 groups. A value of *P* < 0.05 was considered significantly different.

## Additional Information

**How to cite this article**: He, H. *et al.* NK cells promote neutrophil recruitment in the brain during sepsis-induced neuroinflammation. *Sci. Rep.* **6**, 27711; doi: 10.1038/srep27711 (2016).

## Supplementary Material

Supplementary Information
