# Supplementary material for: NK cells promote neutrophil recruitment in the brain during sepsis-induced neuroinflammation
Source: Sci Rep. 2016 Jun 8;6:27711. doi: 10.1038/srep27711 (PMC4897692; doi:10.1038/srep27711)
Supplement: Supplementary Information [file srep27711-s1.pdf]

NK cells promote neutrophil recruitment in the brain during sepsis-induced neuroinflammation

Hao He[1], Tingting Geng[1], Piyun Chen[1], Meixiang Wang[1], Jingxia Hu[1], Li Kang[1], Wengang Song[1, *] & Hua Tang[1, *]

[1]Institute of Immunology, Taishan Medical University, Taian, Shandong, 271000, China

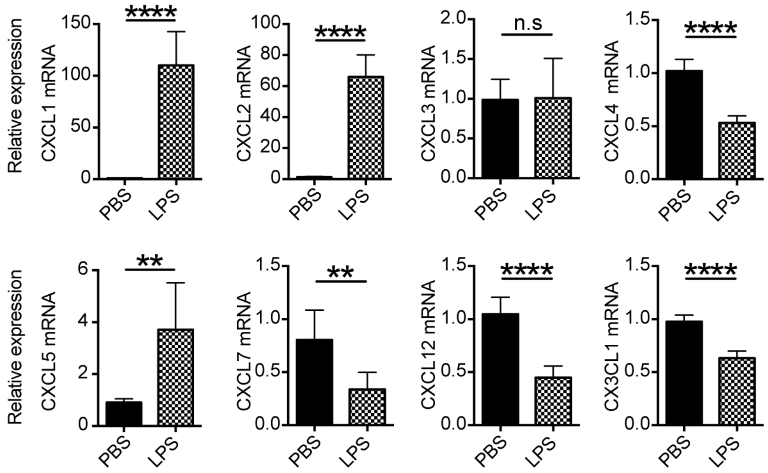

Supplementary Fig. 1. Expression of chemokines for attracting neutrophils in the brain. Twelve hours after LPS or PBS treatment, mRNA (n=6 per group) was extracted from the whole brain of mice. qPCR was performed to detect the expression of chemokines for attracting neutrophils. **P* < 0.05, ***P* < 0.01, ****P* < 0.001, *****P* < 0.0001, ANOVA. Means ± SD are shown. Data shown are representative of at least 2 independent experiments.

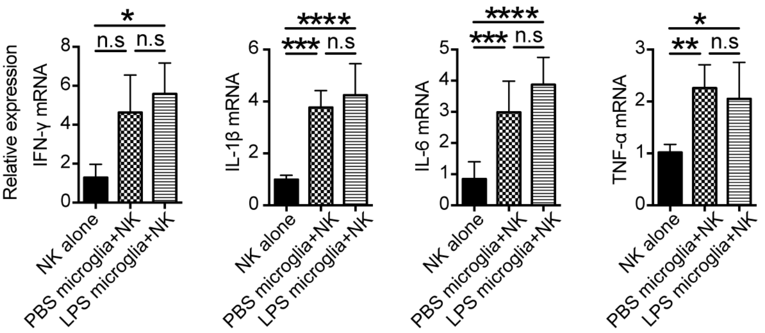

Supplementary Fig. 2. Expression of proinflammatory cytokines by NK cells cocultured with microglia *in vitro*. $CD3^-CD19^-NK1.1^+$ NK cells (1×10^5) sorted from bone marrow in naïve mice were cocultured with or without microglia (2×10^5) sorted from mice treated with PBS or LPS for 3 days. Eleven hours later, NK cells in the coculture were sorted again by flow cytometry for mRNA extraction and subsequent cytokine analysis by qPCR. **P* < 0.05, ***P* < 0.01, ****P* < 0.001, *****P* < 0.0001, ANOVA. Means ± SD are shown. Data shown are representative of at least 2 independent experiments.

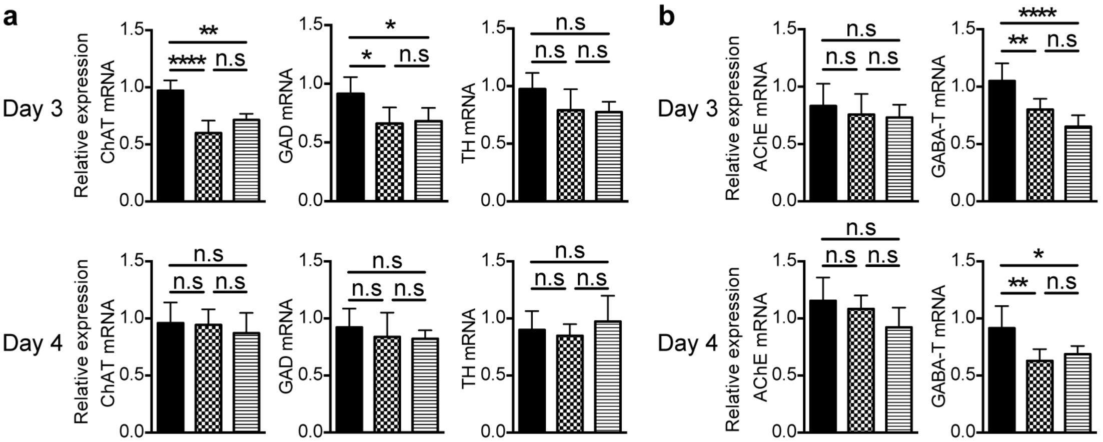

Supplementary Fig. 3. Depletion of NK cells does not regulate the expression of rate-limiting enzymes for synthesis or degradation of acetylcholine, gamma-aminobutyric acid and catecholamine. Mice with or without depletion of NK cells were treated with PBS or LPS for 3 and 4 days. mRNA was extracted from the whole

brain (n=4~6 per group) and used for analysis of the expression of rate-limiting enzymes for neurotransmitter synthesis and degradation by qPCR. (**a**) Choline acetyltransferase (ChAT) is for acetylcholine synthesis; glutamate decarboxylase forward (GAD) is for GABA synthesis; tyrosine hydroxylase (TH) is for catecholamine synthesis; (**b**) Acetylcholinesterase (AChE) is for acetylcholine degradation; GABA-transaminase (GABA-T) is for GABA degradation. **P* < 0.05, ***P* < 0.01, ****P* < 0.001, *****P* < 0.0001, ANOVA. Means ± SD are shown. All data in this figure are representative of at least 2 independent experiments.

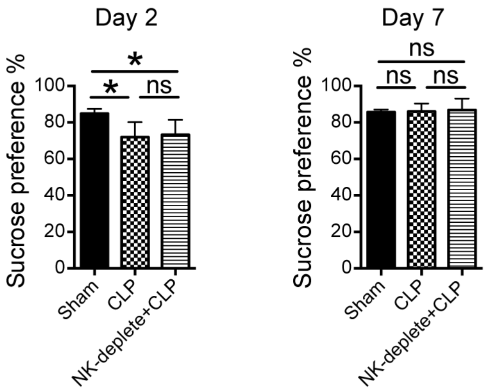

Supplementary Fig. 4. Regulation of depression-like behavior by NK cells in low-grade CLP. Mice with or without depletion of NK cells received surgery and were divided into sham group and low-grade CLP group. On day 2 and day 7, sucrose preference test was performed to evaluate the depression-like behavior of mice from different groups (n=8 per group). **P* < 0.05, ***P* < 0.01, ****P* < 0.001, *****P* < 0.0001, ANOVA. Means ± SD are shown. Data shown are representative of at least 2 independent experiments.

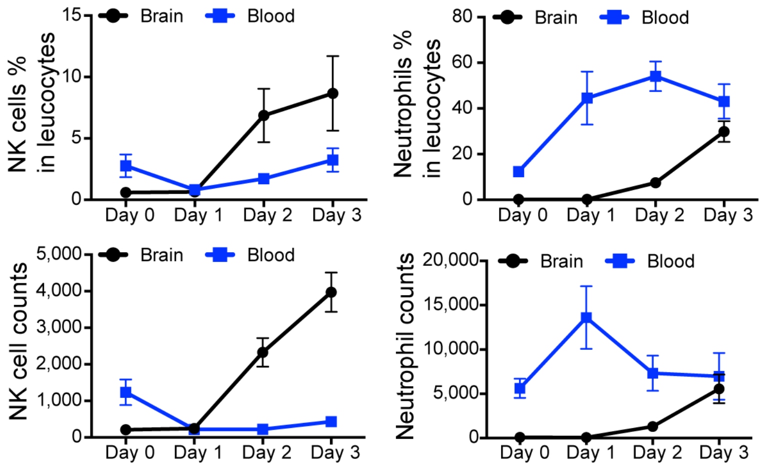

Supplementary Fig. 5. Dynamic changes of NK cells and neutrophils in the blood and brain during LPS-induced inflammation. At indicated time point after LPS treatment, mice (n=4 per group) were killed for assessment of NK cell and neutrophils in the blood and brain. Histogram shows the percentage and cell number of $CD19^-CD3^-NK1.1^+$ NK cells and $CD11b^+Ly6C^-Gr1^+$ neutrophils gated in $CD45^+$ leukocytes.

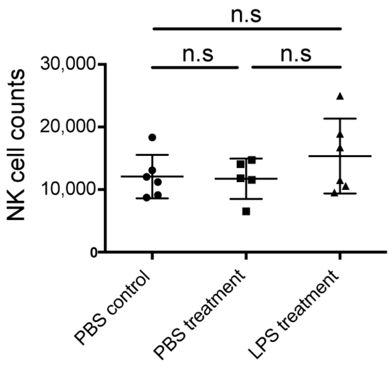

Supplementary Fig. 6. Microglia could not attract NK cells in the early stage of neuroinflammation. Twenty-one hours after PBS or LPS treatment, microglia (8×10^4) were sorted from brain in PBS-treated mice and LPS-treated mice. Then *in vivo* recruitment assay was performed to detect the attraction of NK cells by microglia (n=5~6, per group). ANOVA. Means ± SD are shown. Data shown are representative of at least 2 independent experiments.

Supplementary Table S1. Primers used for qPCR analysis

Gene	Forward Primer	Reverse Primer
Gapdh	TGTGTCCGTCGTGGATCTGA	TTGCTGTTGAAGTCGCAGGAG
Cxcl1	TGCACCCAAACCGAAGTCAT	CTCCGTTACTTGGGGACACC
Cxcl2	CCAACCACCAGGCTACAGG	GCGTCACACTCAAGCTCTG
Cxcl3	GAAAGGAGGAAGCCCCTCAC	ACACATCCAGACACCGTTGG
Cxcl4	TGGTCCCGAAGAAAGCGATG	TTCAGGGTGGCTATGAGCTG
Cxcl5	CACTCGCAGTGGAAAGAACG	CGTGGGTGGAGAGAATCAGC
Cxcl7	ATTGCAACGGAAATCGCCTG	TGTTGCAAAGGTTGCTTGGAA
Cxcl9	GGAGTTCGAGGAACCCTAGTG	GGGATTTGTAGTGGATCGTGC
Cxcl10	CCAAGTGCTGCCGTCATTTTC	GGCTCGCAGGGATGATTTCAA
Cxcl11	GGCTTCCTTATGTTCAAACAGGG	GCCGTTACTCGGGTAAATTACA
Cxcl12	AGAAACCTTCCACCAGAGCAG	GCCGGATCTTGTGTTGAGTGA
Ccl3	TTCTCTGTACCATGACACTCTGC	CGTGGAATCTTCCGGCTGTAG
Ccl4	TTCCTGCTGTTTCTCTTACACCT	CTGTCTGCCTCTTTTGGTCAG
Ccl5	GCTGCTTTGCCTACCTCTCC	TCGAGTGACAAACACGACTGC
Ccl8	TCTACGCAGTGCTTCTTTGCC	AAGGGGGATCTTCAGCTTTAGTA
Cx3cl1	ACGAAATGCGAAATCATGTGC	CTGTGTCGTCTCCAGGACAA
Xcl1	TTTGTCACCAAACGAGGACTAAA	CCAGTCAGGGTTATCGCTGTG
Il-1β	GCAACTGTTCCTGAACTCAACT	ATCTTTTGGGGTCCGTCAACT
Il-6	TCCAGTTGCCTTCTTGGGAC	GTGTAATTAAGCCTCCGACTTG
Tnf-α	ATGTCGGCTCCAGGACCTTA	GGTAGTAACTGTTGACACCCACT
Ifn-γ	ACAGCAAGGCGAAAAAGGATG	TGGTGGACCACTCGGATGA
AChE	ACCGATACTCTGGACGAGGC	CCTGCTTGCTATAGTGGTCG
ChAT	CCATGACTGACCACAAGGCT	TCAATGGCCATGCCGGTTAT
TPH2	CTGAATCCGCCTGAGAGCAT	CCGTACATGAGGACTCGGTG
TH	TACTTTGTGCGCTTCGAGGT	GGAACCTTGTCCTCTCTGGC
GAD	CTGTCCCTGTGTGACAACCA	TGGTAAGCTGCTTTGGCTCG
MAO-A	GCTTATGTGGGACCAACCCA	GGAAATGCACCACGGAATGG
MAO-B	TCCACATTGACCAGACAGGG	CTTCATGCCCAAAGCAGGTG
SERT	GCGACGTGAAGGAAATGCTG	GGAGTTGGGGTGGACTCATC
GABA-T	GAACACTGGGGCTTGGATGA	TTGGCCGAAACTCCTCCTTG

AChE, Acetylcholinesterase; ChAT, choline acetyltransferase; TPH2, Neuronal Tryptophan hydroxylase 2; TH, tyrosine hydroxylase; GAD, Glutamate decarboxylase; MAO-A, Monoamine oxidase A; MAO-B, Monoamine oxidase B;

SERT, serotonin transporter; GABA-T, GABA-transaminase
